# Supplementary figures and images for: Improved patient satisfaction and diagnostic accuracy in skin diseases with a Visual Clinical Decision Support System—A feasibility study with general practitioners
Source: PLoS One. 2020 Jul 29;15(7):e0235410. doi: 10.1371/journal.pone.0235410 (PMC7390264; doi:10.1371/journal.pone.0235410)

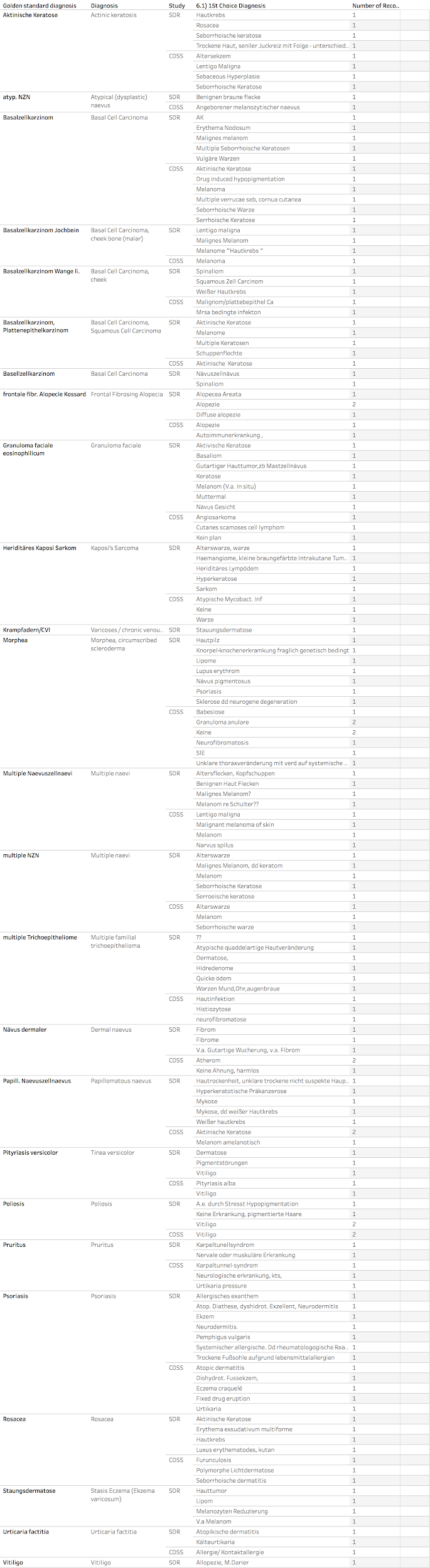

Supplement: S1 Appendix — Overall, the pattern was very heterogeneous using both CDSS or SDR. (TIF) [file pone.0235410.s002.tif]

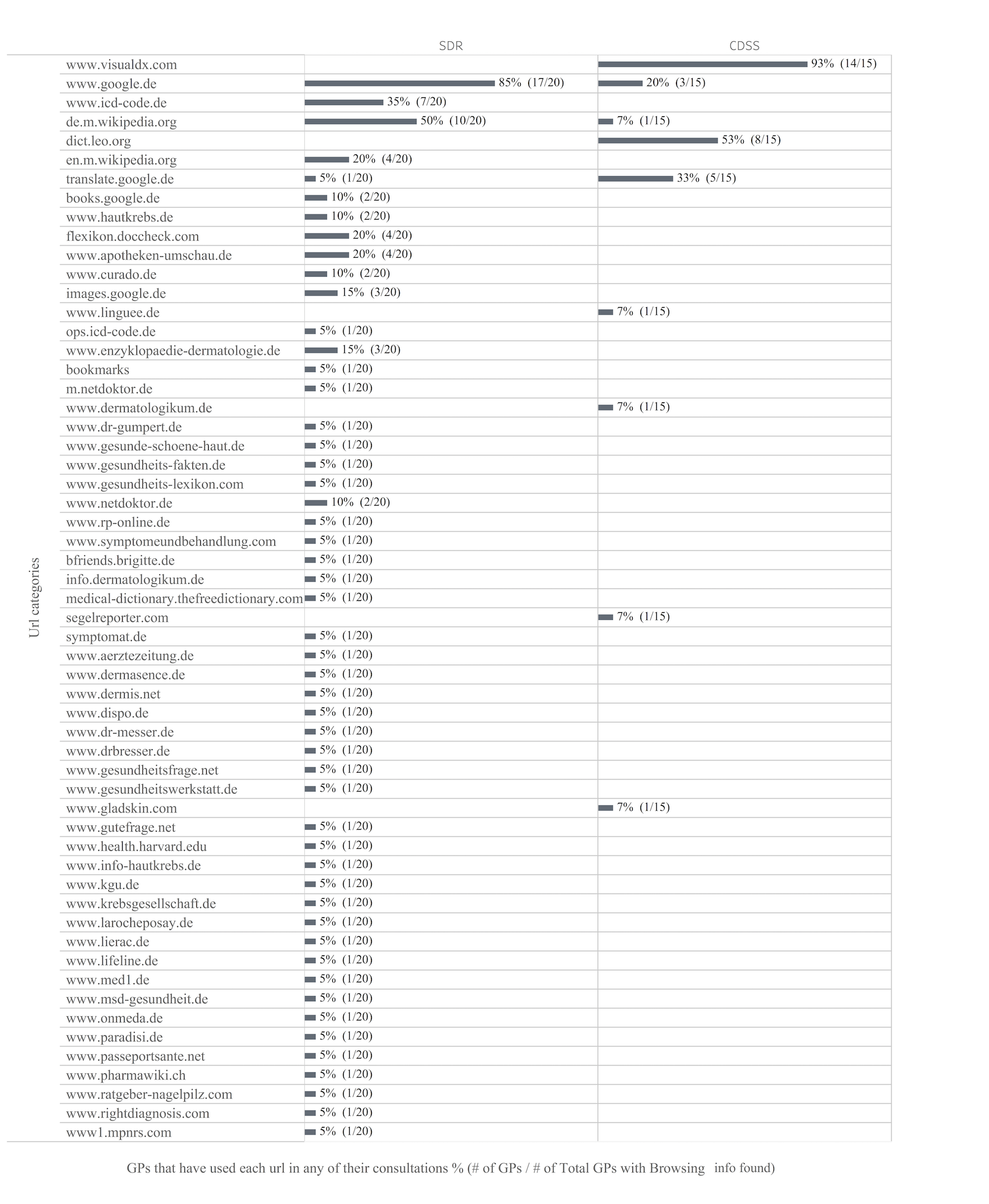

Supplement: S2 Appendix — During each consultation the internet activity was registered. The most frequently used websites in both groups were Google search engine, Wikipedia and Google books. For one GP in the CDSS-arm the browsing history on internet use was not retrievable because the GP used the CDSS application. (TIF) [file pone.0235410.s003.tif]
