# Supplementary material for: Improved patient satisfaction and diagnostic accuracy in skin diseases with a Visual Clinical Decision Support System—A feasibility study with general practitioners
Source: PLoS One. 2020 Jul 29;15(7):e0235410. doi: 10.1371/journal.pone.0235410 (PMC7390264; doi:10.1371/journal.pone.0235410)
Supplement: S1 Table — (DOCX) [file pone.0235410.s004.docx]

| Please read the statements carefully and rate your personal experience during the examination accordingly. Please fill in a patient satisfaction form after each consultation | | |
| --- | --- | --- |
| Question 1 | The doctor gave me as much information as I wanted | 1 Poor  2  3  4  5 Excellent |
| Question 2 | The doctor talked in terms I could understand | 1 Poor  2  3  4  5 Excellent |
| Question 3 | The doctor encouraged me to ask questions | 1 Poor  2  3  4  5 Excellent |
| Question 4 | The doctor involved me in decisions as much as I wanted | 1 Poor  2  3  4  5 Excellent |
| Question 5 | The doctor discussed next steps | 1 Poor  2  3  4  5 Excellent |
| Question 6 | The doctor spent the right amount of time with me | 1 Poor  2  3  4  5 Excellent |
| Question 7 | That the doctor used a textbook or the internet while seeing me | ... bothered me  ...did *not* bother me  I don't know |
| Question 8.1 | The doctor used images to explain my condition | Yes (please answer 8.2)  No |
| Question 8.2 | That the doctor used images to explain my condition made me feel more supported | Yes  No |
| Question 9 | The doctor calmed me down (in case you were worried about your condition) | Yes  No  Not applicable |
| Question 10 | The doctor's diagnosis matches the one that was given to me at the start of the study | Yes  No |
