# Supplementary material for: Improved patient satisfaction and diagnostic accuracy in skin diseases with a Visual Clinical Decision Support System—A feasibility study with general practitioners
Source: PLoS One. 2020 Jul 29;15(7):e0235410. doi: 10.1371/journal.pone.0235410 (PMC7390264; doi:10.1371/journal.pone.0235410)
Supplement: S2 Table — *Required. CDSS: clinical decision support system (n = 16 GPs), SDR: standard (n = 21 GPs). (DOCX) [file pone.0235410.s005.docx]

|  | CDSS | | SDR | | Dermatologist,  Gold standard | |
| --- | --- | --- | --- | --- | --- | --- |
|  | Physician study number* | Please enter | Physician study number* | Please enter |  |  |
|  | Start of examination* | Please enter hh:mm | Start of examination* | Please enter hh:mm |  |  |
| Patient data | Patient study number* | Please enter 2-digit number | Patient study number* | Please enter 2-digit number | Patient study number* | Please enter |
|  |  |  |  |  | Recruitment date* | DD/MM/YYYY |
|  |  |  |  |  | Informed consent signed?* | Yes  No |
|  | Sex* | Female  Male | Sex* | Female  Male | Sex* | Female  Male |
|  | Age* |  | Age* |  | Age* |  |
|  |  |  |  |  | Current occupation | Please enter |
|  |  |  |  |  | Highest level of education | 1) Still in school  2) without lower secondary education  3) Lower secondary education  4) Middle school / high school for 10 to 16 year old students  5) A levels / university entrance certificate 6) Apprenticeship  7) Bachelor’s degree  8) Master’s degree  9) PhD  10) Else |
|  | Chief/presenting complaints*,  Symptoms of patient to seek medical attention | Please enter | Chief/presenting complaints*,  Symptoms of patient to seek medical attention | Please enter |  |  |
|  | For how long has the patient had these symptoms | Please enter (months) | For how long has the patient had these symptoms | Please enter (months) | - | - |
| Diagnoses | 1st choice diagnosis* | Please enter | 1st choice diagnosis* | Please enter | Diagnosis* |  |
|  | ICD-10 code(s) | Please enter | ICD-10 code(s) | Please enter | ICD-10 code(s) of confirmed diagnoses* | Please enter |
|  | 2nd choice diagnosis | Please enter | 2nd choice diagnosis | Please enter | Additional skin diseases | Please enter |
|  | ICD-10 code(s) | Please enter | ICD-10 code(s) | Please enter | ICD-10 code(s) of further diagnoses* | Please enter |
|  | Further testing (only for 1st choice diagnosis)* | Please enter | Further testing (only for 1st choice diagnosis)* | Please enter | Tests that were used for confirmation* | Please enter |
|  | Therapy / medication for treatment plan* (only for 1st choice diagnosis) | Please enter | Therapy / medication for treatment plan* (only for 1st choice diagnosis) | Please enter | Current treatment plan / medications* | Please enter |
|  | Further Comments | Please enter | Further Comments | Please enter |  |  |
|  | Referral* | Yes  No | Referral* | Yes  No | Had the patient first been examined by a GP would referral have been necessary?* | Yes  No |
|  | If yes, to what specialty | Please enter | If yes, to what specialty | Please enter | If yes, to what specialty | Please enter |
|  | If yes, what is the reason for the referral? | Please enter | If yes, what is the reason for the referral? | Please enter |  |  |
|  | End of examination* | Please enter hh:mm | End of examination* | Please enter hh:mm |  |  |
| Supporting tools |  |  | Did you use any tools or support to make this diagnosis?* | Yes  No |  |  |
|  |  |  | If yes, please answer the following questions: Which support did you use? | Medical textbook  Internet  Other |  |  |
|  | How did you apply the information of CDSS to your consultation?* | Diagnosis  Management issue  Clinical manifestation  Cause  Background/ patient question  Other: | How did you apply the information of the tool / support to your consultation? | Diagnosis  Management issue  Clinical manifestation  Cause  Background/ patient question  Other: |  |  |
|  | What was the outcome of the activity?* | Modified treatment plan  Reinforced treatment plan  Increased knowledge  Improved competency  No impact  Other: | What was the outcome of the activity? | Modified treatment plan  Reinforced treatment plan  Increased knowledge  Improved competency  No impact  Other: |  |  |
