# Supplementary material for: Improved patient satisfaction and diagnostic accuracy in skin diseases with a Visual Clinical Decision Support System—A feasibility study with general practitioners
Source: PLoS One. 2020 Jul 29;15(7):e0235410. doi: 10.1371/journal.pone.0235410 (PMC7390264; doi:10.1371/journal.pone.0235410)
Supplement: S3 Table — *P-values were calculated using Mann Whitney U test comparing those giving the right diagnosis with those giving the wrong diagnosis. (DOCX) [file pone.0235410.s006.docx]

|  | **SDR (n=175)** | **CDSS (n=159)** | **Both (n=334)** |
| --- | --- | --- | --- |
| Age | 0.47 | 0.66 | 0.98 |
| Years of practice | 0.32 | 0.84 | 0.92 |
| CDSS practice prior to the study | - | 0.98 | - |
| Duration of consultation | 0.50 | 0.17 | 0.39 |
