# Supplementary material for: Improved patient satisfaction and diagnostic accuracy in skin diseases with a Visual Clinical Decision Support System—A feasibility study with general practitioners
Source: PLoS One. 2020 Jul 29;15(7):e0235410. doi: 10.1371/journal.pone.0235410 (PMC7390264; doi:10.1371/journal.pone.0235410)
Supplement: S1 File — (PDF) [file pone.0235410.s007.pdf]

Patient Satisfaction through Visual Clinical Decision Support System (PSAVIDS)

Principal Investigator (PI) Prof. Dr. Eckhard Breitbart
Association of Dermatological Prevention (ADP)
Am Krankenhaus 1a (ÄTZ), D-21614 Buxtehude
Tel: +49 4161 5547901
info@professor-breitbart.de

Study Manager Dr. Kohelia Choudhury
Association of Dermatological Prevention (ADP)
Cremon 11, 20457 Hamburg
Tel: +49 40 209 131 30
choudhury@euroskin.eu

Sponsor's representative Chief Medical Officer - Dr. John Zibert
LEO Innovation Lab (LEOilab)
1 Niels Hemmingsens Gade, DK-1153 Kbh K
Tel : +45 2291 7020
John@leoilab.com

1. Project Team 6

2. Background and rationale for the study 6

3. Objectives 8

4. Study design 8

4.1 Statement of design 8

4.2 Study population 9

4.3 Sample size 11

4.4 Study location 11

4.5 Study duration 12

4.6 Ethics exemption 12

5. Methodology 12

5.1 Recruitment of physicians 12

5.2 Recruitment of patients 12

5.3 Informed consent 13

5.4 Compensation for participation 13

5.5 Randomization 13

5.6 VisualDx intervention (webinar) 13

5.7 Control arm 14

5.8 Study execution 14

5. 9 Instructions for participants 15

6. Potential risks for participants 15

7. Data confidentiality 16

8. Data collection and evaluation 17

8.1 Data capture 17

8.2 Data management and analysis 18

9. Publication and Dissemination of results 18

10. Time table 19

11. References **20**

12. Attachments **21**

Abbreviations

ADP	Association of Dermatological Prevention e.V., www.unserehaut.de
DOB	Date of birth
DDX	Differential Diagnoses Generator
CRF	Case report form
GP	General Practitioner
KLAS	Provides reviews and reports on vendors of healthcare information technology, www.klasresearch.com
LEOilab	LEO innovation lab, www.leoilab.com
PI	Principal Investigator
PSAVIDS	Patient Satisfaction through Visual Clinical Decision Support System
PT	Patient
RCT	Randomized controlled trial

Figures

Figure 1: PSAVIDS study design - overview

Figure 2: Detailed study procedure of both study arms

Figure 3: Study execution phase

Figure 4: PSAVIDS time table

Table of Contents

1. Project Team

2. Background and rationale for the study

3. Objectives

4. Study design

4.1 Statement of design
4.2 Study population
4.3 Sample size
4.4 Study location
4.5 Study duration
4.6 Ethics exemption

5. Methodology

5.1 Recruitment of physicians
5.2 Recruitment of patients
5.3 Informed consent
5.4 Compensation for participation
5.5 Randomization
5.6 VisualDx intervention (webinar)
5.7 Control arm
5.8 Study execution
5. 9 Instructions for participants

6. Potential risks for participants

7. Data confidentiality

8. Data collection and evaluation

8.1 Data capture
8.2 Data management and analysis

9. Publication and Dissemination of results

10. Time table

11. References

12. Attachments

1. Project Team

Institution	Name	Email
Association of Dermatological Prevention (ADP)	Prof. Dr. Eckhard Breitbart, PI	info@professor-breitbart.de
	Dr. Marianne Breitbart	
	Kohelia Choudhury, PhD study manager	choudhury@euroskin.eu
	Susanne Fengler MSc PH HP, team member	fengler@unserehaut.de
	Henriette Bunde, team member	bunde@unserehaut.de
	Vroni Deichmann, team member	weber@professor-breitbart.de
LEO Innovation Lab (Leoilab)	CMO Dr. John Zibert	John@leoilab.com
	Daniel Dickens	dickens.daniel@gmail.com
VisualDX	Anne Komanecky Executive Director Business Development	akomanecky@visualdx.com
LEO Pharma	Dr. Hans Joachim Hutt	HJHDE@leo-pharma.com

2. Background and rationale for the study

Differential diagnosis generator (DDX)

VisualDx is a computer-assisted differential diagnosis tool with over 40,000 images of medical conditions, most related to dermatology. It can be accessed using mobile technology such as smartphones and tablet computers. Unlike textbooks indexed by disease, it allows physicians to enter patient descriptions like age, sex, and symptoms. The tool rapidly generates images of potential diagnoses that mostly match the entered criteria, enabling physicians to "rule in or out" by comparing a patient's skin condition with the images. Furthermore, it also provides concise disease-specific information as well as information on management, therapy, and handouts for patients. The content is written, reviewed, and frequently updated by more than 100 experts in the fields of medicine (Tleyjeh et al. 2006, VisualDx 2016). Thus, VisualDx gives information at the point-of-care and can assist clinicians with diagnosis, treatment, patient communication, and self-education.

First launched in 2001, VisualDx was designed by dermatologists for non-dermatologists such as general practitioners (GP), emergency care clinicians, and public health professionals, with the aim of improving diagnostic accuracy of skin conditions and reducing misdiagnosis-related harms. In a randomized controlled trial (RCT) it was demonstrated that the use of VisualDx by non-dermatologists improves the diagnostic accuracy of skin complaints compared with text book use (Papier et al., 2001). Results of another study show

that VisualDx has the potential to assist primary care physicians in correctly diagnosing cellulitis, a commonly misdiagnosed condition (David et al., 2011).

A survey among VisualDx users carried out by KLAS in 2012 revealed that 94% of the users agreed that VisualDx is able to confirm a diagnosis, 70% of users said it influences therapy decisions, and 85% of users think that the tool improves patient interaction as physicians share images of different conditions during consultation (Almacen, 2014). Furthermore, use of this real-time decision support results in shorter consultation lengths, and thus, saves clinicians' and patients' time (VisualDX, 2016).

In the U.S. the Institute of Medicine recommends the use of healthcare IT resources, such as VisualDx and to date it is has been licensed in over 1500 hospitals and large clinics worldwide. More than 85 medical schools in the U.S. are using this tool to teach students how to quickly build a differential diagnosis. VisualDx promotes lifelong learning based on information-assisted decision-making rather on memory-based education.

<u>Patient satisfaction</u>
Nowadays, patients are considered as consumers of health services, thus the assessment of their perspective has become a priority in medical management (Cimas et al, 2016). Furthermore, the shift to a biopsychosocial model of healthcare and patient-centered care has emphasized the patient's part in medical decision making and promoted shared-decision making (Clayman et al, 2016). Evidence suggests that when patients participate in medical decision-making they are more satisfied with their care, which can result in higher compliance to health regimens and better health outcomes (Greenfield et al., 1985, Kaplan et al., 1989, Suh and Lee, 2010). Patient satisfaction is therefore a common indicator for quality healthcare.

<u>Rationale for the study</u>
Diagnostic accuracy and user satisfaction are usually the main focus in studies on differential diagnoses generators (DDX), including VisualDx, whereas patients' perspective is often not investigated. Despite the widespread use of VisualDx there is no data showing how the tool affects patient satisfaction. As an important indicator for quality healthcare, patient satisfaction and/or patient engagement should be included in those kinds of studies.

VisualDx has the potential to improve the quality of consultations by (i) engaging patients in the decision-making process, e.g. sharing images and information reassures patients and builds confidence in the doctor's diagnosis, (ii) reducing the length of consultations, and (iii) increasing the diagnostic accuracy.
We will conduct a feasibility study to assess patient satisfaction as well as lengths of consultation and diagnostic accuracy of VisualDx consultations. The *<u>Pa</u>tient <u>S</u>atisfaction through <u>Vi</u>sual Clinical <u>D</u>ecision <u>S</u>upport System* (PSAVIDS) study takes place in Germany. Patients with skin diseases that are first diagnosed by experienced dermatologists (gold

standard) will subsequently be examined by a group of GPs using VisualDx. A week later the same cohort of patients will be examined by another group of GPs without the use of VisualDx or other DDX. GPs are randomly assigned to one of the groups. We will analyze and compare above mentioned outcome measures of both groups.

To our knowledge this is the first study investigating the impact of VisualDx on patient satisfaction. Moreover, this feasibility study will also reveal whether the research design and implementation is practical, thus it will set the foundation of future large scale RCTs.

3. Objectives

The overall purpose of this study is to investigate whether the use of VisualDx by GPs improves the quality of skin complaints consultations in terms of patient satisfaction, consultation length, and diagnostic accuracy compared with standard medical consultations.

Primary objective:

- To directly compare the patient-satisfaction scores of VisualDx consultations with standard medical consultations.

Secondary objectives:

- To directly compare the consultation lengths of VisualDx consultations with standard medical consultations.
- To evaluate the diagnostic accuracy of VisualDx consultations against a gold standard and to compare it with the diagnostic accuracy of standard medical consultations.

4. Study design

4.1 Statement of design

In order to investigate the impact of VisualDx consultations on patient satisfaction, consultation length, and diagnostic accuracy, a feasibility study will be conducted. Patients with confirmed skin disorders (chronic and newly diagnosed) will be recruited by two dermatologists from their own pool of patients. In addition, GPs will be enrolled and randomized to the VisualDx arm and control arm, respectively. The so called ‘test patients’ will then be examined by physicians in the VisualDx arm as well as in the Control arm (standard medical consultations; **Figure 1**). Patient satisfaction in both arms will be assessed after each examination by questionnaires, while case report forms (CRF) will be used to evaluate consultation lengths and diagnostic accuracy.

Figure 1: PSAVIDS study design - overview

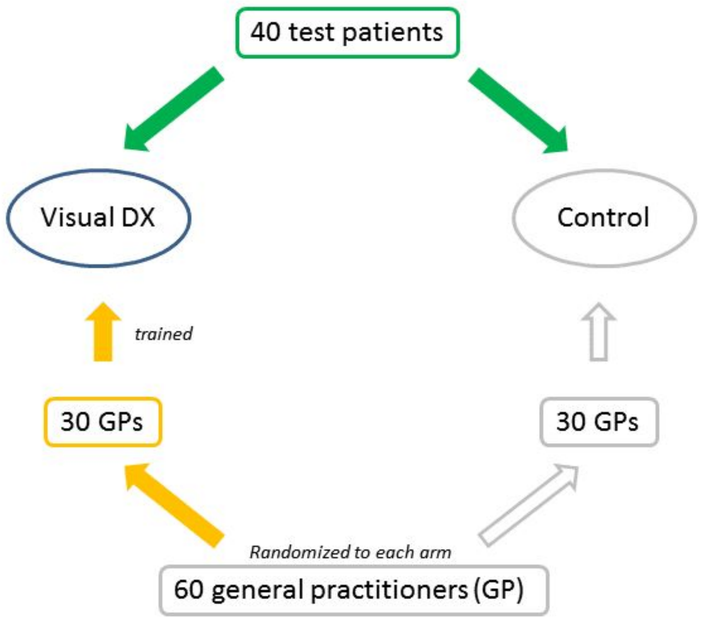

4.2 Study population

1.) Test patients

Inclusion criteria (ALL criteria must be met)

- Male and female German residents ≥ 18 years of age
- Suffering from skin disease/s (chronic and/or newly diagnosed)
- Skin diseases must be confirmed by two dermatologists - gold standard
- Skin diseases should not change significantly over the study period (to ensure that data is comparable)

To ensure a mix of complexity of the cases, a broad range of skin conditions will be included.

2.) Physicians

Inclusion criteria (ALL criteria must be met)

- Practice–based GPs
- At least completion of residency
- Must read and understand English in order to use VisualDx
- Must be accustomed to the use of Internet and mobile technology (smart phone or tablet computer)
- Applies only to GPs in the VisualDx arm: completion of the VisualDx webinar

Exclusion criteria (one of the following criteria)

- Previous use of an electronic differential builder (e.g. UpToDate, DynaMed, MCConsult, MediBox)
- Extra education / special interest in dermatology
- Medical students

4.3 Sample size

1.) *Test patients:* 40 patients with confirmed skin diseases will be recruited.
2.) *Physicians:* 60 GPs will be enrolled.
3.) *Number of examinations: 480*
    Each test patient is examined by 6 GPs, i.e. 6 diagnoses per patient, resulting in a total of 240 diagnoses per study arm (**Figure 2**). A total of 480 patient satisfaction questionnaires and diagnoses, respectively, will be analyzed.

Figure 2: Detailed study procedure of both study arms

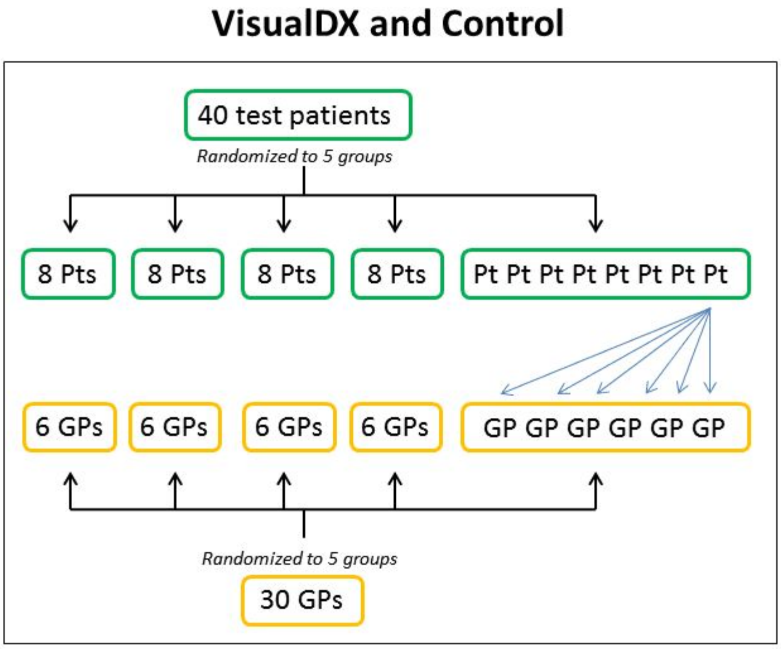

Power calculation; the sample size is determined to be minimum 150 diagnoses for each group, based on the two independent groups of GPs, where outcome measures was based on a diagnostic accuracy in SDR and CDSS of 45∓5%, and 65∓5%, with an alpha of 0.05 resulted in a power calculation of 100%.

4.4 Study location

The PSAVIDS study takes place centralized either in Buxtehude or in Hamburg, Germany. In Buxtehude both study arms can be carried out in the PI's practice (Prof. Dr. Eckhard Breitbart, Am Krankenhaus 1a (ÄTZ), 21614 Buxtehude) and/ or in the Dermatology

Department of the adjacent hospital (Elbekliniken-Buxtehude, Am Krankenhaus 1a, 21614 Buxtehude).

4.5 Study duration

Total study duration: 7 months (see Figure 4)

Preparation phase: 3 months

- Ethics waiver
- Agreement on study location
- Approval of study protocol by LEOilab
- Recruitment of 60 GPs and 40 test patients
- Designing the VisualDx webinars

Study execution phase: 1 month (**Figure 3)**

- Hosting two VisualDx webinars (2 weeks)
- VisualDx arm execution (1 week)
- Control arm execution (1 week)

Evaluation phase: 3 months

- Data analyses (patient questionnaires and CRFs)
- Approval of final report by LEOilab
- Publication

4.6 Ethics exemption

Since the PSAVIDS does not pose any health and psychological risks to test patients and GPs (see 6. Potential risks for participants), it is anticipated that this study does not need ethics approval from an Institutional Review Board. Proof of waiver: informal email.

5. Methodology

5.1 Recruitment of physicians

Recruitment of GPs will be carried out in cooperation with the Bezirks Landesärztekammer Stade (Regional medical association of Stade, Germany).

5.2 Recruitment of patients

40 test patients will be recruited by two experienced dermatologists (Drs Breitbart) from their own pool of patients. The test patients need to meet ALL inclusion criteria (4.2 Study population). To include a broad range of skin diseases, patients with 'easy to diagnose' skin conditions (e.g. atopic dermatitis, psoriasis, pityriasis rosea) and more 'difficult to diagnose' skin conditions (e.g. basal cell carcinoma, erythema nodosum, nodular vasculitis) will be

recruited. Care will be taken to balance the proportions of 'easy to diagnose' and 'difficult to diagnose' skin conditions.

At recruitment a gold standard CRF for each enrolled patient will be completed by the dermatologists to confirm eligibility and to collect baseline data. These CRFs will be used as references (see 8.1 Data capture) for the evaluation of the diagnostic accuracy. Each CRF will include a photo of the skin disease taken at the time of recruitment.

Photos will be taken at recruitment, on the VisualDx execution day, and on the control arm execution day. This will help to document changes that might appear to skin lesions/conditions during the preparation phase and study execution phase (internal control).

5.3 Informed consent

Written consent will be obtained from each test patient prior to study entry. The dermatologists will fully inform test patients about the study objectives, the design, the VisualDx intervention and about any risks that are associated with it (6. Potential risks for participants). Furthermore, it will be explained how confidentiality of data will be maintained, especially with respect to the information about the participant which would otherwise be known only to the dermatologist, but in this case will be made known to the study team. Test patients will be asked to give their consent for their data to be included in a research database (see 8.1 Data capture). The dermatologist has to mention the participant's right to withdraw from the study at any time. The patient has the option NOT to participate and it will be explained that nonparticipation has no effect on the future doctor-patient relationship.

5.4 Compensation for participation

1.) *Test patients*

Recruited test patients will receive financial compensation for each study day (250€/day) and for their travel costs (up to 50€/person/day). The total amount will be wire transferred to patients only after completion of the 2nd study execution day.

2.) *Physicians*

Enrolled physicians will receive financial compensation for the study day (500€) and for their travel costs (up to 50€/person).

5.5 Randomization

Upon enrollment, 60 GPs will be allocated at random to the VisualDx arm and the control arm. Names of the physicians will be thrown in a hat with the first 30 drawn out allocated to the VisualDx arm and the remainder to the control arm. This random allocation minimizes selection bias and the effect of possible confounders.

5.6 VisualDx intervention (webinar)

GPs (n=30) randomized to the VisualDx arm will receive an invitation for a training webinar that will be hosted by VisualDx representatives. Attendance is mandatory, therefore two dates will be offered two weeks in advance of the study (**Figure 3**). The aim of this webinar is to train GPs how to build a differential diagnosis with VisualDx and to demonstrate how patients can be engaged. In addition, the study aims, the design and execution will be explained briefly.

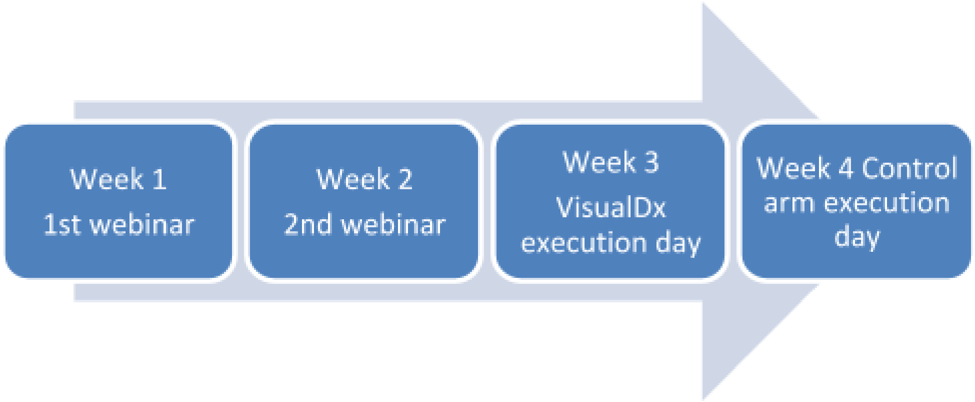

Figure 3 : Study execution phase

Each GP will receive a tablet computer and his individual VisualDx log in details by mail prior to the training webinars. The tablet computer will have the VisualDx application installed. Although VisualDx has been described as intuitive to use, GPs will be encouraged to use the tool in their practice in advance of the study to gain confidence in building differential diagnoses. They will have 1 to 2 weeks for training and practicing (**Figure 3)** . Papier et al. (2001) showed that with minimal training (10 minutes) non-dermatologists were able to effectively use the tool, so 1 week minimum for practicing should be sufficient.

5.7 Control arm

On the Control arm execution day each GP (n=30) will receive a tablet computer with Internet access to complete the electronic CRFs. They are allowed to use the Internet and their own textbooks as support tools for their standard medical consultations. This will mimic 'real world' situations. However, access to the VisualDx application as well as to similar applications (e.g. UpToDate, DynaMed, MCConsult, MediBox) and websites will be blocked.

5.8 Study execution

The PSAVIDS study will take place over two consecutive Saturdays. First the VisualDx arm will be executed. 40 test patients will be randomized to 5 blocks of 8 patients (**Figure 2**), while 30 GPs will be randomized to 5 blocks of 6 physicians. Each patient will then be examined by 6 GPs. Immediately after each consultation patients need to complete the patient satisfaction questionnaire.

This procedure will be repeated on the following Saturday for the Control arm. The same set of test patients but different physicians will participate. It is estimated that a ‘full visit’ takes approximately 20 minutes (10 minutes consultation, 5 minutes questionnaire, 5 minutes break). In addition, on each study execution day photos of the skin conditions will be taken before the consultations start (see 0).

5. 9 Instructions for participants

The following instructions will be given to participants to ensure a smooth study execution:

1) *Test patients*
    - A clinician or medical assistant takes a photo of the skin disease at the beginning of each study day.
    - Patients are requested not to disclose any information regarding their skin disease to the GPs during the consultations, i.e. the correct diagnosis, any treatment plans, and advice and information they received from their dermatologists. Furthermore, patients are encouraged to act as if they were new patients with an undiagnosed skin disease.
    - After each consultation patients need to complete the electronic 'patient satisfaction questionnaire' which takes maximum 5 minutes.

2) *Physicians*
    - GPs have maximum 10 minutes for a consultation (completion of CRF included)
    - Each consultation will be documented on an electronic CRF.
    - GPs are encouraged to act as having 'real world' consultations, which includes:
        - history taking,
        - stating a presumptive diagnosis,
        - explaining further tests,
        - treatment options and plans,
        - making a referral when necessary,
        - answering questions from patients, and
        - giving information.
    - On the VisualDx execution day, GPs will use the tablet computers they received for training. GPs will use VisualDx to go straight to a known diagnosis page to use images and information to educate the patient or will use VisualDx to build a differential diagnosis. It is not mandatory to build a differential diagnosis if the diagnosis is known to the GP. They will be encouraged to use VisualDx to educate the patient with images and information. The tablet computers will only grant access to VisualDx, to the electronic CRFs, and to an English-German dictionary; access to other websites will be denied.
    - Control arm: for the standard medical consultations, GPs are allowed to use supporting tools as described in 5.7 Control arm.

6. Potential risks for participants

1.) Test patients

There are hardly any risks involved for test patients. Only patients with a confirmed skin disease will be enrolled in this study. Apart from a pain-free visual inspection of the skin, *no subsequent invasive procedures and tests and no medications are given to the patients.* These patients are already seeing a dermatologist and some will be receiving medication. Therefore, anxiety and distress patients often feel when getting a new diagnosis and/or a treatment plan is very unlikely to occur.

The only discomfort and embarrassment that patients might feel arises from the fact that some have to get undressed for the examination.

2.) *Physicians*

Risks to GPs taking part in the study have not been identified.

7. Data confidentiality

1.) Test patients

All information about participation in this study will be kept confidential. The dermatologists will keep the signed informed consent forms. Each test patient will be given unique patient study number. The CRFs will contain personal data such as DOB, sex, and the unique patient study number. The patient questionnaire carries only the unique patient study number. Clearly identifiable information such as name, address, and insurance number will not be submitted to the study team and will stay with the dermatologists. Data analyses will be carried out only with anonymized data.

2.) Physicians

All information about participation in this study will be kept confidential. Each GP will receive a unique physician study number which will be documented on the CRFs. Patient questionnaires will not contain any physician identifiable data. Data collected on physician's individual performance will never be exposed. Results on diagnostic accuracy and consultation lengths will be published only in aggregate form and no individual be it patient or physician can ever be identified from research findings.

However, if a physician requests information on his individual performance, it will be disclosed to him.

8. Data collection and evaluation

8.1 Data capture

Four data capture instruments will be used: 1) Patient satisfaction questionnaire, 2) CRFs, 3) internet history of tablet computers, and 4) VisualDX usage logs.

1.) Patient satisfaction questionnaire

Patients will complete electronic patient satisfaction questionnaires on *Google Forms.* The questionnaires have been developed according to the Communication Assessment Tool (Makoul et al, 2007) which has been developed after the SEGUE Framework (Makoul et al, 2001). Patients are asked to assess: Their status of being informed about their condition

- How well they understood the GP
- If they could ask questions
- If they were involved in decisions
- If the GP discussed the next steps
- If the time of the consultation was sufficient
- If they were bothered by the usage of Visual DX or in the Control arm if the GP used other tools
- If the GP used images to explain the condition
- If the GP calmed the patient down
- If the diagnosis told to the patient, was the same as written down on the CRF
- If the diagnosis matched the one from the gold-standard examination

2.) GPs in both study arms will complete electronic CRFs on *Google Forms* which is a HIPAA compliant survey tool. The CRFs will capture following data:
   - Patient’s DOB, sex, and chief complaint
   - Length of consultation
   - ICD-10 code of presumptive diagnosis
   - Recommended tests, medications/treatment plans, referrals
   - Any tools (internet, text books) that were used
   - Impact on the consultation that the tool had

At the time of patient recruitment, dermatologists will document following information also using *Google Forms* (gold standard CRF):

- Date of recruitment
- Patient’s DOB and sex
- Occupation and highest education level
- Chief complaint
- ICD-10 code of confirmed diagnosis

- Tests that were used for confirmation (e.g. histopathology)
- Medications/treatment plans that the patient is receiving

3.) The Internet history of tablet computers used in both study arms will be analyzed and documented in Excel. This will reveal the type of support tools that were used by physicians.

4.) VisualDx usage logs will show which findings or symptoms and diagnoses were entered and viewed by GPs. These information will be transferred to Excel and analyzed.

8.2 Data management and analysis

Data in *Google Forms* (CRF, Patient satisfaction questionnaire) will be saved in CVS format and exported to Excel. Data will be stored in unchanged form (raw data set). Raw data will be reviewed to identify any discrepancies (incomplete data, illogical data, and illegible data) and plausibility checks will be conducted. After the database has been cleaned, it will be stored and locked. For statistical analyses the 'cleaned' database will be exported into SPSS 21.

Outcomes measures

The unique patient study numbers will be used to link datasets, e.g. the gold standard CRF with VisualDx CRF and with the control CRF.

1.) Patient satisfaction scores; direct comparison of scores of both study arms
2.) Length of consultation: direct comparison of the median and average consultation length (minutes) of both study arms
3.) Diagnostic accuracy
    - VisualDx diagnoses compared to gold standard diagnoses: number of correct diagnoses
    - Control arm diagnoses compared to gold standard diagnoses: number of correct diagnoses

9. Publication and Dissemination of results

All efforts will be made to publish the results of this study in peer-reviewed scientific journals with consent of all research partners and participating organizations. Journals of interest would be family medicine journals, dermatology journals, and public health journals.

Dissemination of results to participants will take place via email and mail. In addition, participants will be made aware of the results if requested.

10. Time table

Figure 4: PSAVIDS time table

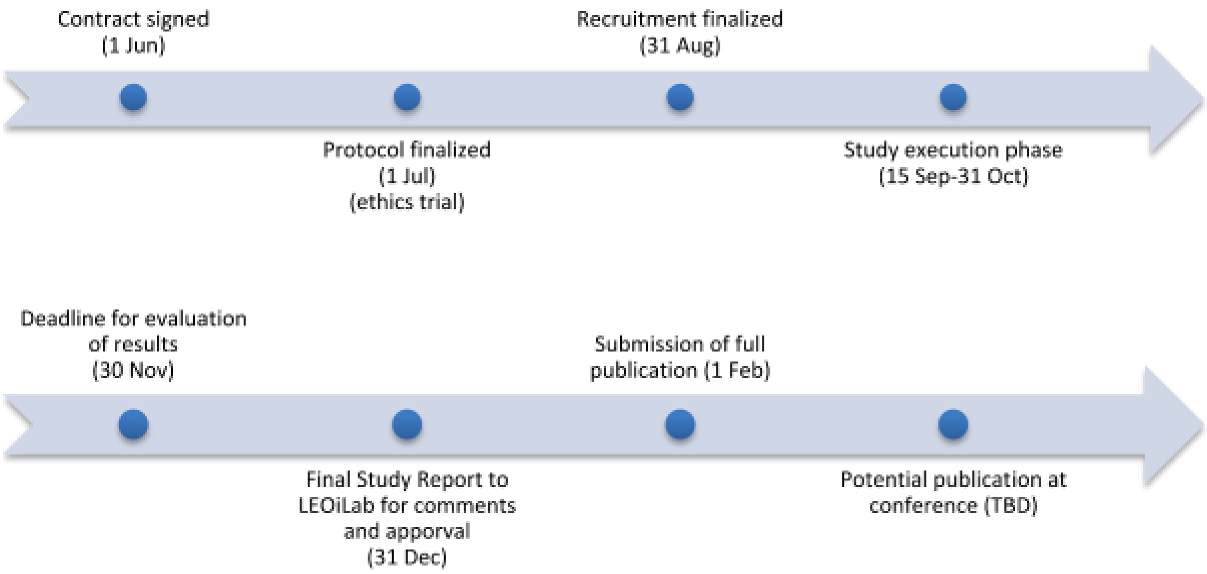

11. References

Almacen MC (2014) VisualDx Clinical Decision Support System – ACDSS Research: retrieved from: https://www.google.de/url?sa=t&rct=j&q=&esrc=s&source=web&cd=1&cad=rja&uact=8&ved=0ahUKEwj4rqqS1MDNAhXLCsAKHayuCAcQFggnMAA&url=http%3A%2F%2Fmariaa-northwesternu.weebly.com%2Fuploads%2F3%2F1%2F7%2F1%2F31716081%2Fsession_4_almacenmaria_406dl_visual_dxcdss_homework_final.docx&usg=AFQjCNH-q7SM3RxjKTDQQK9KAVqJz6dlbg

Cimas, M., Ayala, A., Garcia-Perez, S., Sarria-Sanatmera, A. and Forjaz, M.J. (2016) The patient satisfaction questionnaire of EUprimecare project: measurement properties *Intern. Journal for Quality in Health Care Advance Access* 1-6.

Clayman, M., Bylund, C., Chewning, B., Makoul, G. (2016) The impact of patient participation in health decisions within medical encounters: A systematic review *Journal of Medical Decision Making*36:427-452.

David CV, Chira S, Eells SJ, Ladrigan M, Papier A, Miller LG, Craft N. (2011) Diagnostic accuracy in patients admitted to hospitals with cellulitis. *Dermatol Online J. 2011 Mar 15;17(3):1.*

Greenfield S, Kaplan S, Ware JE Jr. (1985) Expanding patient involvement in care. Effects on patient outcomes *Ann Intern Med 1985; 102(4):520-8*

Kaplan SH, Greenfield S, Ware JE Jr. (1989) Assessing the effects of physician-patient interactions on the outcomes of chronic disease *Med Care 1989; 27(3 Suppl):S110-27*

Makoul, G., Krupat, E. and Chang, C.-H. (2007) Measuring patient views of physician communication skills: Development and testing of the Communication Assessment Tool *Journal of Patient Education and Counseling* 67:333-342.

Makoul, G. (2001) The SEGUE Framework for teaching and assessing communication skills *Journal of Patient Education and Counseling* 45:23-34.

Papier A, Allen E, McDermott M. (2001) Visual informatics: real-time visual decision support. Poster presented at: American Medical Informatics Association 2001 Annual Symposium; November 3-7, 2001; Washington, DC.

Tleyjeh IM, Nada H, Baddour LM. (2006) VisualDx: decision-support software for the diagnosis and management of dermatologic disorders. *Clin Infect Dis. 2006 Nov 1;43(9):1177-84.*

VisualDX 2016: retrieved from http://www.visualdx.com

Suh WS and Lee CK (2010) Impact of shared-decision making on patient satisfaction *Journal of Preventive Medicine and Public Health 2010;43(1): 26-34*

12. Attachments

- Patient satisfaction questionnaire – Control group
- Patient satisfaction questionnaire – VisualDx group
- CRF – Control group
- CRF – VisualDx group
- CRF – Gold standard
-

PSAVIDS Study

* Required

Patient Satisfaction Form Control

Please read the statements carefully and rate your personal experience during the examination accordingly. Please fill in a patient satisfaction form after each consultation.

Patient study number *

Your answer

Please fill in the date of your consultation *

Date

1. The doctor gave me as much information as I wanted *

poor

1
2
3
4
5

excellent

2. The doctor talked in terms I could understand *

poor

1
2
3
4
5

excellent

3. The doctor encouraged me to ask questions *

poor

1
2
3
4
5

excellent

4. The doctor involved me in decisions as much as I wanted *

poor

1
2
3
4
5

excellent

5. The doctor discussed next steps *

poor

1
2
3
4
5

excellent

6. The doctor spent the right amount of time with me *

poor

1
2
3
4
5

excellent

7. That the doctor used a textbook or the internet while seeing me... *

...did bother me
...did not bother me
I don't know

8.1 The doctor used images to explain my condition *

yes (please answer 8.2)
no

8.2 That the doctor used images to explain my condition made me feel supported more

yes
no

9. The doctor calmed me down (in case you were worried about your condition)

yes
no
Not applicable

10. The diagnosis that the doctor told me correlated with the one that was written down by him/her. *

yes
no
I don't know

11. The doctor's diagnosis matches the one that was given to me at the start of the study *

yes
no

SUBMIT

PSAVIDS Study

* Required

Patient Satisfaction Form Visual DX

Please read the statements carefully and rate your personal experience during the examinations accordingly. Please fill in a patient satisfaction form after each consultation.

Patient study number *

Your answer

Please fill in the date of your examination *

Date

1. The doctor gave me as much information as I wanted *

poor

1
2
3
4
5

excellent

2. The doctor talked in terms I could understand *

poor

1
2
3
4
5

excellent

3. The doctor encouraged me to ask questions *

poor

1
2
3
4
5

excellent

4. The doctor involved me in decisions as much as I wanted *

poor

1

2
3
4
5
excellent

5. The doctor discussed next steps *

poor
1
2
3
4
5
excellent

6.The doctor spent the right amount of time with me *

poor
1
2
3
4
5
excellent

7. That the doctor used Visual DX while seeing me... *

...did bother me
...did not bother me
I don't know

8.1 The doctor used images to explain my condition *

yes (If yes, please answer 8.2)
no

8.2 That the doctor used images to explain my condition made me feel supported more

yes
no

9. The doctor calmed me down (in case you were worried about your condition)

yes
no
Not applicable

10. The diagnosis that the doctor told me correlated with the one that was written down by him/her. *

yes

no

11. The doctor's diagnosis matches the one that was given to me at the start of the study *

yes

no

Formularbeginn

PSAVIDS - Control CRF

Date of study execution

*Required

Physician study number *

Formularende

Start of examination *

Please enter hh:mm

Time :

Patient data

1) Patient study number *

Please enter 2-digit number

2) Sex *

Female

Male

3) Date of birth *

DD / MM / YYYY

4) Chief/presenting complaints *

Symptoms of patient to seek medical attention

Diagnoses

1.1) 1st choice diagnosis *

1.2) 2nd choice diagnosis

2) ICD-10 code(s) *

3) Further testing (only for 1st choice diagnosis)

4) Therapy / medication for treatment plan * (only for 1st choice diagnosis)

5) Further Comments

6.1) Referral *

Yes

No

6.2) If yes, to what specialty

7) End of examination *

Please enter hh:mm

Time :

Supporting tools

8.1) Did you use any tools or support to make this diagnosis? *

Yes

No

8.2) If yes, please answer the following questions: Which support did you use?

Medical text book

Internet

Other:

8.3) How did you apply the information of the tool / support to your consultation?

Diagnosis

Management issue

Clinical manifestation

Cause

Background/ patient question

Other:

8.4) What was the outcome of the activity?

Modified treatment plan

Reinforced treatment plan

Increased knowledge

Improved competency

No impact

Other:

Formularbeginn

Formularbeginn

PSAVIDS - VisualDX CRF

Date of study execution

*Required

Physician study number *

Formularende

Start of examination *

Please enter hh:mm

Time :

Patient data

1) Patient study number *

Please enter 2-digit number

2) Sex *

Female

Male

3) Date of birth *

DD / MM / YYYY

4) Chief/presenting complaints *

Symptoms of patient to seek medical attention

Diagnoses

1.1) 1st choice diagnosis *

1.2) 2nd choice diagnosis

2) ICD-10 code(s) *

3) Further testing (only for 1st choice diagnosis)

4) Therapy / medication for treatment plan * (only for 1st choice diagnosis)

5) Further Comments

6.1) Referral *

Yes

No

6.2) If yes, to what specialty

7) End of examination *

Please enter hh:mm

Time :

8) How did you apply the information of VisualDx to your consultation? *

Diagnosis

Management issue

Clinical manifestation

Cause

Background/ patient question

Other:

9) What was the outcome of the activity? *

Modified treatment plan

Reinforced treatment plan

Increased knowledge

Improved competency

No impact

Other:

Formularbeginn

Formularbeginn

PSAVIDS - gold standard CRF

To be completed by Drs. Breitbart

*Required

1) Patient study number *

Formularende

Recruitment date *

DD / MM / YYYY

2) Informed consent signed *

yes

no

Date of birth *

DD / MM / YYYY

3) Sex *

Female

Male

4) Current occupation

5) Highest level of education

Option 1

Option 2

Option 3

Option 4

Other:

6) Diagnosis *

7) Additional skin diseases

8) ICD-10 code(s) of confirmed diagnoses *

9) Tests that were used for confirmation *

10) Current treatment plan / medications *

Formularbeginn
