# Supplementary material for: Improved patient satisfaction and diagnostic accuracy in skin diseases with a Visual Clinical Decision Support System—A feasibility study with general practitioners
Source: PLoS One. 2020 Jul 29;15(7):e0235410. doi: 10.1371/journal.pone.0235410 (PMC7390264; doi:10.1371/journal.pone.0235410)
Supplement: Attachment — Submitted filename: Response to Reviewers PONE-D-19-34156.docx [file pone.0235410.s008.docx]

## Rebuttal letter PONE-D-19-34156

-------------------------------------------------------------------------------------------------------------------------

Additional Editor Comments (if provided):

Before considering publication of your work, major revisions should be made.

See below:

Reviewer #1: This study is about patients’ perspectives on the use of CDSS (VisualDx) by GPs on diagnosing dermatologic condition compared to standard consultation. The authors claim that the use of CDSS improves patient satisfaction, improves diagnostic accuracy and reduces referrals to specialists.

While the study explores noble concept, the language is unclear in some parts, making it difficult to follow. I advise the authors to work on improving the flow and readability of the text.

Thank you for acknowledging that the focus of the manuscript has merits. According to the reviewer’s advice we have now worked on the flow and readability throughout the entire manuscript. We believe that this has overall strengthened the manuscript and made it more concise and easier to follow.

All references to line numbers in the following are based on the 'Revised Manuscript with Track Changes'. For our replies we have used blue font, and sentences that refer directly to wordings in the manuscript have been written in blue bold font.

1. Foremost, the study does not appear to be sound. The study design is questionable and may not measure what the authors are trying to do. The patients knew their diagnosis prior to being evaluated by either CDSS or SDR, which may affect how they perceive the visits/satisfaction score.
   a. Yes, it is true that patients knew their diagnosis beforehand, however some patients didn’t remember it even though. Nevertheless, this information should not affect how they perceive one type of visit over another, and in addition there was one week between visits. As such, we believe it is relevant to make the comparison between the SDR and CDSS-visits. An alternative study design is hard to imagine as fluctuation of skin characteristics over time is in this setup standardized to one week, and otherwise would be questionable.
2. The authors mention that the objectives of the study are to assess patient satisfaction, diagnostic accuracy and length of consultation, but in conclusion (especially in abstract), they mention patient satisfaction, diagnostic accuracy and number of referrals. I recommend that the authors answer the questions initially intended to answer.
   a. Thank you for pointing out this inconsistency. We have now removed this and addressed the duration of consultations as appropriate
3. The authors say, “Other important aspects of quality healthcare are diagnostic accuracy and length of consultations (line 56).” Please include the evidence that supports this statement.
   a. Thank you for asking for references here. References have been added, and furthermore clarity has been added. The sentence will now read:
      **“Other important aspects of quality healthcare may be diagnostic accuracy and length of consultations in order to achieve clinical effectiveness and patient safety.”** (Line 58)
      Reference:
      Kirch, W., Shapiro, F. & Fölsch, U.R. Health care quality: Misdiagnosis at a university hospital in five medical eras. J Public Health 12, 154–161 (2004)

      Natasha Elmore, Jenni Burt, Gary Abel, Frances A Maratos, Jane Montague, John Campbell, Martin Roland: Investigating the relationship between consultation length and patient experience: a cross-sectional study in primary care. British Journal of General Practice 66 (653): e896-e903 (2016).
4. The authors mention 2 studies that showed the diagnostic accuracy of CDSS, so it is unclear what the study’s objective on diagnostic accuracy adds to existing literature.
   a. Thank you for addressing this point. Clearly, this should be sufficiently explained in the manuscript. What clearly separates this study from the previous ones is that we specifically considered a wide range of dermatoses which GPs are likely to encounter in their everyday practice. As such, we aim to reflect a broader/real-world situation with the current study.
      i. We have added the following sentence in the introduction line (76): “**To our knowledge, no studies have previously investigated how this can impact diagnostic accuracy in a group of patients with a range of more complex dermatoses.**”
5. It is unclear how the consent process was taken place. I suggest that the authors include details of the recruitment process of the GPs. Was there any compensation for the participants (patients, GPs, dermatologist)? Also it is unclear if the patients were seen at GPs practice or at a different office, maybe in a research lab?
   a. Thank you for the comments we have the following response:
      i. The recruitment process of GPs is mentioned in the last section of “Study design and population” and was done in collaboration with “Regional Medical Association of Stade, Germany”.
      ii. All patients, GPs, and dermatologists participating were reimbursed in order to compensate travel costs and lost work hours in order to participate in the study.
          This will be reflected in the “Financial Support/Funding statement:”
           “**ADP did according to a fair market value assessment and law and regulations reimburse patients, GP’s and dermatologists for travel expenses and lost work hours.** ” (line 20)
      iii. All in-clinic GP and dermatologist evaluations were conducted at Arbeitsgemeinschaft Dermatologische Prävention (ADP) in Buxtehude. This will be reflected in the beginning of the section “Study design and population”. “**The study was a randomized feasibility study conducted in a dermatology clinic at Hautarztpraxis Buxtehude, Buxtehude, Germany**.” (line 87)
6. The authors mention that the GPs were randomized to SDR vs CDSS, but based on their personal availability. This does not seem to fit the randomization process. Also unclear if the GPs knew which day was CDSS day and which day was SDR day prior to selecting the study day.
   a. We have now clarified this, and the sentence now reads: **“Although GPs were initially randomised to either the CDSS or SDR arm, a few were unavailable on the scheduled study day, and were therefore transferred to the other study arm based on their availability. The GPs were not aware of which study day was the CDSS or the SDR day, respectively, and this was therefore not a part of such a decision.”(line 164)**
7. On line 113, the authors mention that the first 6 questions test patient’s actual satisfaction, but per the reference 10, the questionnaire measures patient view of physician communication skills, which is not synonymous to patient satisfaction.
   a. Thank you for pointing this out, as this was maybe not clearly described. The tool has been specifically developed to capture how patients experience and *perceive* the encounter with their doctor, and the first 6 items more specifically address their satisfaction with this experience.
      i. We’ve changed the following sentence “**The first six questions were regarding the patient’s actual satisfaction with the consultation**.” to **“The first six questions were reflecting patients general satisfaction level, and were constructed to assess how they had experienced the interpersonal communication with the GP during the consultation, to which they should assign a score from 1 (poor) to 5 (excellent).”** (line 122).
8. On line 231, the authors mention, “the relative difference between the patient’s understanding of their diagnosis and the one provided during the consultation was 22% higher in the CDSS….” The statement is unclear as written, and makes it sound like there were more diagnostic discrepancies in CDSS arm. Also the figure number should be 3B instead of 2B.
   a. Thanks for pointing this out. Agreed that this was unclear.
      i. We’ve replaced the sentence with the following: **“Also, in the CDSS relative to the SDR arm the patients more frequently (+22%) replied that the diagnosis they had been given by the GP at the end of the consultation matched that provided by the dermatologist prior to the study (Fig 3B, P=0.034)”** (line 241).
      ii. As such, we have now also corrected the figure number to 3B
9. The authors claim that there were no statistically significant differences among GPs in CDSS vs SDR on diagnostic accuracy. It is unclear if that is the case in each dermatologic condition since only 3-8 GPs evaluated each patient, not all GPs. Subgroup sample size may be too small to support that the diagnostic accuracy was due to CDSS use.
   a. It is a bit unclear exactly what the reviewer refers to, but seemingly it is the mentioning that the diagnostic accuracy was not impacted by the differences in age, and years of experience in medical practice. This statistic has been applied across all SDR and CDSS consultations, respectively, and thereby reflect a crude overall comparison across the different skin conditions. As also shown in figure 2, the different diagnoses have been evenly distributed to the two study arms. Within the 175 (CDSS) + 159 (SDR) consultations and accompanying diagnoses, we investigated if those that arrived at the correct diagnosis were older or more experienced. Our data suggest that this is not the case. However, it is correct that we cannot imply anything in detail about how this is reflected for the individual diagnoses, and we also do not intend to do so.
10. The authors mention tablet (in method section), internet, and iPad (results and discussion sections). It is unclear if they all mean the same thing for the purpose of this study. Did the GPs have access to internet via some other means other than a tablet/iPad? Did GPs in SDR arm use tablet/iPad for any other use besides internet access?
    a. This is a good point, and we haven’t been sufficiently clear about this:
       i. The GPs did only have access to the tablets in both arms.
       ii. In the SDR arm the GPs could access the internet on the tablet or use textbooks they brought themselves.
       iii. In the CDSS arm they were asked to use the CDSS, but did have access to the internet on the tablet, the latter was rarely used.
       iv. We have now updated the method section (line 92-97), and streamlined the manuscript reflecting “Tablet” and not iPad.
11. The statement on line 291 says, “We found that 52% of the patients in the CDSS arm compared with only 39% in the SDR…..” However, according to figure, it is 39% and 23%, respectively.
    a. Thank you for highlighting this, that is a typo. We’ve now corrected this in the manuscript (line 312)
12. On line 302, the authors argue that the negative correlation between overall satisfaction with the consultation and the length of the consultation was only evident in the CDSS arm where the GPs were using an iPAD. It is unclear if the GPs in SDR arm were also using iPad or not. In method section, the authors mention that both groups were provided with a tablet.
    a. Thank you for this comment, both groups were provided a tablet. We have now updated the method section (Line 92-97).
13. The last paragraph of the discussion section, the authors makes comments that are not the main objective of the study or supported by other studies. For instance, the authors say that CDSS can help achieve patient outcome and save money for healthcare system, but based on the most recent systemic review, there was no clear evidence that CDSS improves patient outcome or is cost effectiveness. (Black AD, Car J, Pagliari C, et al. The impact of eHealth on the quality and safety of health care: a systematic overview. PLoS Med. 2011;8(1):e1000387. Published 2011 Jan 18. doi:10.1371/journal.pmed.1000387
    a. It is true that we discuss potential implications of increasing diagnostic accuracy and patient satisfaction. In that sense this is more a speculation about the perspectives for healthcare systems. We have rephrased to make it clear that this is speculations, and have also included the reference provided by the reviewer. (Line 381)
       “**However, it should be mentioned that the overall cost-effectiveness of implementing different eHEALTH-solutions in the healthcare system is still being debated (Black AD et al.).**”
    b. In addition we have deleted the following sentence; “...likely to lead to improved adherence, increased quality of life and overall improved quality of life and/or happiness, translating into more work days and increased productivity of the society at large”. (Line 387-389)
14. On line 335, the authors mention that the GPs in CDSS arm felt more confident with the diagnosis that they chose not to refer the patients. The questionnaire did not explore the GPs perspectives on how confident they felt, so we cannot jump to conclusion that they felt more confident based on the number of referrals placed. The reason for referral could be diagnostic, but also therapeutic.
    a. Agreed on this point, thank you. Implying that the lack of referral from the doctors is because the GPs feel more confident is an interpretation, because we did not directly ask the GPs about this. Consequently, we have removed this interpretation from the text which now reads as follows; **“It was interesting to note that by using the CDSS more GPs chose not to refer the patient, irrespective of whether the diagnoses turned out to be right or not.” (**line 373**)**
15. The authors’ last comment on the discussion section (increased quality of life, happiness, increased productivity of the society) is too broad, and does not seem immediately relevant to the study objectives nor supported by the study.
    a. Agreed. We have deleted this part.
16. The authors should include the limitation of the study (such as not being a randomized controlled trial, etc) to keep in mind the generalizability of the study.
    a. Yes, this is a good point. We have now included a mentioning of the limitations of the study (line 360-367)

**Journal Requirements:**

When submitting your revision, we need you to address these additional requirements.

1. Please ensure that your manuscript meets PLOS ONE's style requirements, including those for file naming. The PLOS ONE style templates can be found at

<http://www.journals.plos.org/plosone/s/file?id=wjVg/PLOSOne_formatting_sample_main_body.pdf> and <http://www.journals.plos.org/plosone/s/file?id=ba62/PLOSOne_formatting_sample_title_authors_affiliations.pdf>

2. Please provide additional details regarding participant consent. In the ethics statement in the Methods and online submission information, please ensure that you have specified whether consent was suitably informed.
   a. Thank you for highlighting this, the methods section has now been updated: “**Following verbal information given by the dermatologists, written consent was obtained from each test patient prior to study entry.**” (line 90)
3. We note that you have indicated that data from this study are available upon request. PLOS only allows data to be available upon request if there are legal or ethical restrictions on sharing data publicly. For information on unacceptable data access restrictions, please see <http://journals.plos.org/plosone/s/data-availability#loc-unacceptable-data-access-restrictions>.
   a. We will not share data that conflicts with subject identity (patients, GPs, dermatologists). We will also follow the PLOS guidelines on data availability.

In your revised cover letter, please address the following prompts:

- If there are ethical or legal restrictions on sharing a de-identified data set, please explain them in detail (e.g., data contain potentially identifying or sensitive patient information) and who has imposed them (e.g., an ethics committee). Please also provide contact information for a data access committee, ethics committee, or other institutional body to which data requests may be sent.
  - We will only share de-identified data sets as per approved legal assessment per case. This will be stated in the cover letter.
- If there are no restrictions, please upload the minimal anonymized data set necessary to replicate your study findings as either Supporting Information files or to a stable, public repository and provide us with the relevant URLs, DOIs, or accession numbers. Please see <http://www.bmj.com/content/340/bmj.c181.long> for guidelines on how to de-identify and prepare clinical data for publication. For a list of acceptable repositories, please see <http://journals.plos.org/plosone/s/data-availability#loc-recommended-repositories>.
  - In the supporting information we have shared large parts of the study outcomes. This will be stated in the cover letter.

We will update your Data Availability statement on your behalf to reflect the information you provide.

4. Please include captions for your Supporting Information files at the end of your manuscript, and update any in-text citations to match accordingly. Please see our Supporting Information guidelines for more information: <http://journals.plos.org/plosone/s/supporting-information>.

-----------------------------------------------------------------------------------------------------------------------

Reviewers' comments:

Reviewer's Responses to Questions

**Comments to the Author**

1. Is the manuscript technically sound, and do the data support the conclusions?

The manuscript must describe a technically sound piece of scientific research with data that supports the conclusions. Experiments must have been conducted rigorously, with appropriate controls, replication, and sample sizes. The conclusions must be drawn appropriately based on the data presented.

Reviewer #1: Partly

2. Has the statistical analysis been performed appropriately and rigorously?

Reviewer #1: I Don't Know

3. Have the authors made all data underlying the findings in their manuscript fully available?

The [PLOS Data policy](http://www.plosone.org/static/policies.action#sharing) requires authors to make all data underlying the findings described in their manuscript fully available without restriction, with rare exception (please refer to the Data Availability Statement in the manuscript PDF file). The data should be provided as part of the manuscript or its supporting information, or deposited to a public repository. For example, in addition to summary statistics, the data points behind means, medians and variance measures should be available. If there are restrictions on publicly sharing data—e.g. participant privacy or use of data from a third party—those must be specified.

Reviewer #1: Yes

4. Is the manuscript presented in an intelligible fashion and written in standard English?

PLOS ONE does not copyedit accepted manuscripts, so the language in submitted articles must be clear, correct, and unambiguous. Any typographical or grammatical errors should be corrected at revision, so please note any specific errors here.

Reviewer #1: No

5. Review Comments to the Author

Please use the space provided to explain your answers to the questions above. You may also include additional comments for the author, including concerns about dual publication, research ethics, or publication ethics. (Please upload your review as an attachment if it exceeds 20,000 characters)

Reviewer #1: This study is about patients’ perspectives on the use of CDSS (VisualDx) by GPs on diagnosing dermatologic condition compared to standard consultation. The authors claim that the use of CDSS improves patient satisfaction, improves diagnostic accuracy and reduces referrals to specialists….

…

...

__________________________________________________________________________

6. PLOS authors have the option to publish the peer review history of their article ([what does this mean?](https://journals.plos.org/plosone/s/editorial-and-peer-review-process#loc-peer-review-history)). If published, this will include your full peer review and any attached files.

If you choose “no”, your identity will remain anonymous but your review may still be made public.

**Do you want your identity to be public for this peer review?** For information about this choice, including consent withdrawal, please see our [Privacy Policy](https://www.plos.org/privacy-policy).

Reviewer #1: No

[NOTE: If reviewer comments were submitted as an attachment file, they will be attached to this email and accessible via the submission site. Please log into your account, locate the manuscript record, and check for the action link "View Attachments". If this link does not appear, there are no attachment files to be viewed.]

While revising your submission, please upload your figure files to the Preflight Analysis and Conversion Engine (PACE) digital diagnostic tool, <https://pacev2.apexcovantage.com/>. PACE helps ensure that figures meet PLOS requirements. To use PACE, you must first register as a user. Registration is free. Then, login and navigate to the UPLOAD tab, where you will find detailed instructions on how to use the tool. If you encounter any issues or have any questions when using PACE, please email us at figures@plos.org. Please note that Supporting Information files do not need this step.
